# Prevalence of Postoperative Micronutrient Deficiencies in Bariatric Surgery Patients Who Use Transdermal Patches for Supplementation: A Pilot Study

**DOI:** 10.7759/cureus.25989

**Published:** 2022-06-16

**Authors:** Tyler Culpepper, Tamara Lux, Sunny Trivedi, Dan Neal, Kyle Hazen, Max Fleisher, Ronny Samra, Crystal Johnson-Mann, Jeffrey Friedman

**Affiliations:** 1 Department of Medicine, University of Florida, Gainesville, USA; 2 Department of Surgery, University of Florida, Gainesville, USA

**Keywords:** nutritional deficiencies, postoperative complications, multivitamin patch, transdermal vitamins, bariatric surgery

## Abstract

Background

Patients require vitamin and mineral supplementation after bariatric surgery to prevent the development of micronutrient deficiencies. Consuming oral supplements is challenging due to gastric volume restriction. A transdermal patch dosage form may provide adequate micronutrient supplementation without pill burden. The study aims to determine the percentage of patients who have two or more nutritional deficiencies one year after surgery and to determine serum nutrient concentrations and gastrointestinal symptoms over time.

Methods

Patients who planned to undergo bariatric surgery and preferred transdermal patches versus oral supplements were recruited during preoperative office visits. Enrolled patients were instructed to use a transdermal multivitamin patch as per the manufacturer’s instructions. Serum nutrient concentrations and Gastrointestinal Symptom Response Scale scores were determined at baseline and three months, six months, and one year after surgery.

Results

Ninety-two participants completed the study protocol. Twenty-five participants had a full panel of study labs one year after surgery. Among these patients, 19% had two or more micronutrient deficiencies. Vitamin D was the most common deficiency followed by vitamin B6; however, median serum concentrations of both nutrients increased over time. Vitamin B1, folate, and zinc deficiencies were also observed. There were no changes in gastrointestinal symptoms.

Conclusions

Additional studies, including randomized controlled trials, are required to determine if the PatchMD Multivitamin Plus patch (Pilot Rd. STE. B, Las Vegas) can provide adequate supplementation of vitamins and minerals. The patch was not associated with changes in gastrointestinal symptoms.

## Introduction

Over 250,000 bariatric procedures, including laparoscopic sleeve gastrectomy (SG) and Roux-en-Y gastric bypasses (RYGBs), are performed in the United States yearly [1]. Patients who undergo bariatric surgery and are not instructed regarding micronutrient supplement intake develop varying numbers and degrees of deficiencies [2-3]. Some patients develop deficiencies despite having reported oral supplement intake. Aarts et al. demonstrated that up to 43% of their study population developed at least one micronutrient deficiency post SG when instructed to take a standard amount of multivitamins daily [4]. Patients who undergo RYGB may be at greater risk for developing deficiencies [5]. A study in which all patients were prescribed oral multivitamins showed that over one-third of patients continued to be deficient in at least one micronutrient three months after RYGB, and this increased to nearly 100% after 24 months [6]. Vitamin D has been shown to be the most common deficiency one year after bariatric surgery, with other notably prevalent deficiencies in ferritin, folate, and vitamin B12 [7].

Consuming the added volume of oral supplements in addition to the fluid and protein requirements and home medications in the initial postoperative period may be challenging. Dehydration is a leading cause of postoperative emergency department visits and readmissions [8]. Furthermore, undesirable side effects (e.g., constipation from oral iron supplements) potentially lead to nonadherence with supplements [9-10]. Nonadherence may result in vitamin deficiencies. Thiamine deficiencies, for example, can cause constipation as well as symptoms of Wernicke’s encephalopathy, which can be permanent [11-12].

The use of transdermal patches for vitamin D supplementation has been demonstrated in animal studies and a single randomized controlled trial [13-14]. McCormick et al. showed that using a patch for iron supplementation was inferior to oral supplements but led to fewer reported side effects [15]. The effectiveness of patches may be nutrient and dose-dependent but may serve as a superior alternative to oral supplements due to fewer potential gastrointestinal side effects.

The purpose of this pilot study is to determine which and how many nutritional deficiencies patients undergoing bariatric surgery develop postoperatively when they use vitamin patches for supplementation. The primary outcome is the percentage of patients who have two or more nutritional deficiencies one year after surgery. Secondary outcomes include individual serum nutrient concentrations and gastrointestinal symptoms over time. We hypothesize that supplementation with a transdermal patch will prevent the development of both nutritional deficiencies and gastrointestinal symptoms, particularly constipation, postoperatively.

## Materials and methods

Adult participants were recruited from a single center between December 2017 and May 2020. During preoperative office visits, patients who planned to use transdermal patches for vitamin supplementation after surgery were screened. Patients must have been 18 years or older, candidates for an SG or RYGB, able to provide informed consent in English, and able to commit to the one-year study period. Exclusion criteria included planned bariatric revision surgery, presence of a left ventricular assistance device, end-stage renal disease, mutations in the methylenetetrahydrofolate reductase gene, and medical conditions requiring micronutrient supplementation. Informed consent was obtained from all individual participants included in the study and all study procedures were in accordance with the ethical standards of the institutional review board (IRB201701809). The study was preregistered on clinicaltrials.gov (NCT03360435) prior to conducting the research and adheres to the disclosure requirements of the institutional registry (https://clinicaltrials.gov/ct2/show/NCT03360435?titles=Transdermal&cntry=US&state=US%3AFL&city=Gainesville&draw=2&rank=1).

After providing consent, all participants were instructed to purchase and use the Patch MD™ MultiVitamin Plus patch (Pilot Rd. STE. B, Las Vegas) for one year and to apply one patch per day for eight hours as per the manufacturer’s instructions. The micronutrient composition of the patch is provided in Table 1. Participants were asked to avoid taking supplements containing calcium, zinc, copper, iron, B vitamins, and vitamin D. Follow-up appointments were scheduled for three, six, and 12 months after surgery.

**Table 1:** Nutrient composition of the PatchMD Multivitamin Plus patch IU=international units, N/A=not applicable PatchMD Multivitamin Plus patch: Pilot Rd. STE. B, Las Vegas

Micronutrient	Amount	Percent daily value
Vitamin A (as beta-carotene)	10000 IU	200%
Vitamin C (as ascorbic acid)	1000 mg	1667%
Vitamin D3 (as cholecalciferol)	5000 IU	1250%
Vitamin E (as d-alpha tocopherol)	200 IU	667%
Vitamin K2 (as menaquinone-7)	160 mcg	200%
Vitamin B1 (as thiamine mononitrate)	25 mg	1667%
Vitamin B2 (as riboflavin)	25 mg	1471%
Vitamin B3 (as niacin)	40 mg	200%
Vitamin B6 (as pyridoxine HCl)	25 mg	1250%
Folate	400 mcg	100%
Vitamin B12 (as methylcobalamin)	1000 mcg	16667%
Biotin	600 mcg	200%
Pantothenic acid (as calcium D-pantothenate)	25 mg	250%
Calcium (as calcium carbonate)	1500 mg	50%
Iron (as iron bisglycinate)	45 mg	250%
Phosphorus (as tricalcium phosphate)	100 mg	10%
Iodine (as potassium iodide)	150 mcg	100%
Magnesium (as magnesium oxide)	500 mg	125%
Zinc (as zinc oxide)	15 mg	100%
Selenium (as l-selenomethionine)	100 mcg	100%
Copper (as copper gluconate)	2 mg	100%
Manganese (as manganese citrate)	4 mg	200%
Chromium (as chromium picolinate)	200 mcg	167%
Molybdenum (as sodium molybdate)	100 mcg	133%
Potassium (as potassium chloride)	99 mg	3%
Chloride	70mg	2%
Boron (as boron amino acid chelate)	3 mg	N/A

The Gastrointestinal Symptoms Response Scale (GSRS) was administered to each participant to establish a preoperative baseline [16]. During follow-up visits, serum labs were ordered, and participants were asked to complete another GSRS. Study labs included a complete metabolic panel, zinc, copper, iron, ferritin, total iron-binding capacity, vitamin B1, vitamin B6, folate, vitamin B12, and vitamin D. Reference ranges for normal study lab values are shown in Table 2.

**Table 2:** Normal reference ranges for serum nutrient concentrations

Nutrient	Range
Vitamin B1	70 – 180 nmol/L
Vitamin B6	20 – 125 nmol/L
Vitamin B12	180 – 914 pg/mL
Folate	>5.8 ng/mL
Vitamin D total	>/= 30 ng/mL
Zinc	60 – 120 ug/dL
Calcium	8.4 – 10.2 mg/dL
Copper	80 – 155 ug/dL
Iron	30 – 160 ug/dL
Ferritin	11 – 307 ng/mL
Total Iron Binding Capacity	225 – 430 mcg/dL

Participants with serum concentrations less than the normal range were considered deficient. Lab results were obtained at the specified time points +/- 45 days (with the exception of baseline labs, which were obtained up to six months before surgery). Adherence was assessed during follow-up visits and was defined as the use of the patch for at least five days per week. Analyses were done on an intent-to-treat basis.

Mixed-effects linear models were used to assess the changes in serum concentrations across four time points: preop and three, six, and 12 months postop. A separate model was used for each concentration. All models included a fixed factor for time (considered as a categorical variable) and a random factor for subject, to account for the clustering of observations in patients. The global p-value for time is reported for all models. When the global p-value is <.05, p-values for all pairwise comparisons between time points with p<.05 are reported. All analyses were performed using the R statistical software package (V.4.1.1, The R Foundation for Statistical Computing, Vienna, Austria).

## Results

**Figure 1:**
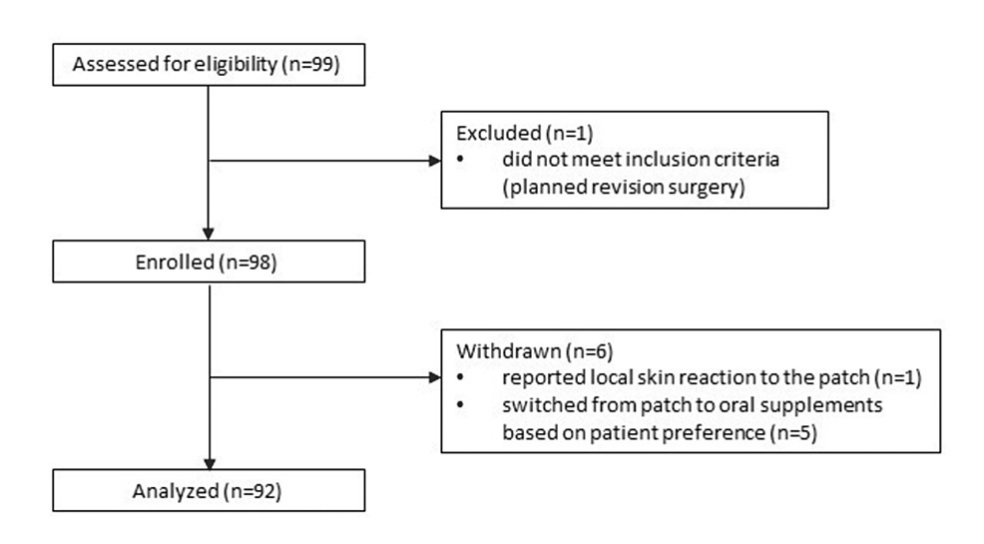
Flow diagram for recruitment, enrollment, and retention of study subjects

Ninety-two participants completed the study protocol. Six participants were withdrawn (Figure 1). Eighty-two participants (88%) were female. The mean and standard deviation for age at the time of consent were 42.2 and 11.7 years, respectively (median 40, range 18 - 87 years). The mean and standard deviation for BMI at the time of consent was 47.5 and 8.4 kg/m, respectively (median 46.0, range 35.2 - 74.5 kg/m). Forty-seven patients underwent SG and 45 patients underwent RYGB.

One year after bariatric surgery, 47 participants had serum nutrient data available; however, only 25 participants had results for all study labs. Among these 25 participants, eight (32%) had at least two nutritional deficiencies. Among all 47 participants, nine (19%) had two or more nutritional deficiencies. One participant had five deficiencies (B1, B6, folate, vitamin D, and Zinc), one participant had four deficiencies (B6, folate, vitamin D, and zinc), two participants had three deficiencies (B6, folate, and vitamin D for both participants), and four participants had two deficiencies (B6 and vitamin D for two participants, vitamin B6 and folate for one participant, and vitamin B6 and vitamin D for one participant).

**Figure 2:**
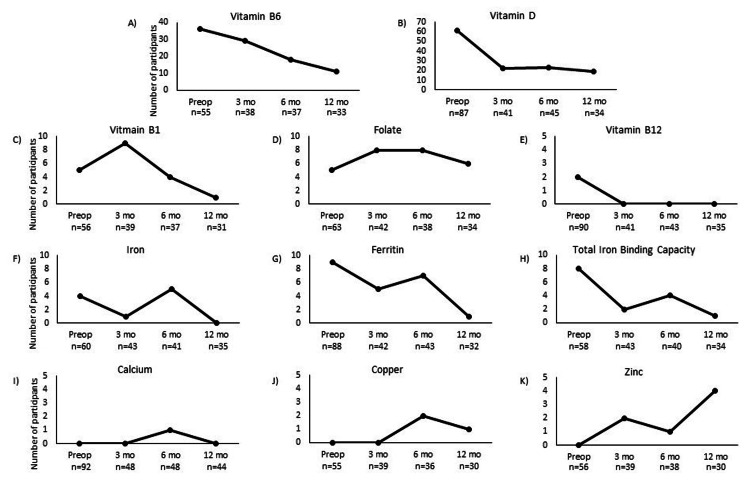
Numbers of participants with individual serum nutrient deficiencies preoperatively and at three, six, and 12-month follow-up A) vitamin B6, B) vitamin D, C) vitamin B1, D) folate, E), vitamin B12, F) iron, G) ferritin, H) total iron-binding capacity, I) calcium, J) copper, K) zinc The n’s represent the number of participants who had serum nutrient data available at each time point. Preop=peroperative, mo=month

The most common deficiency among all 47 participants one year after surgery was vitamin D (n=19), followed by vitamin B6 (n=11); however, this was an improvement from baseline. Figure 2 displays the numbers of patients with deficiencies for each nutrient at each time point. Measures of central tendency for each nutrient concentration at each time point are shown in Table 3. Median serum vitamin D concentrations were significantly higher at all postoperative time points when compared to baseline values. Vitamin B6 was significantly higher 12 months postoperatively, whereas calcium was significantly lower 12 months postoperatively compared to all other time points, but all median serum concentrations were within normal limits. Vitamin B1 was significantly lower three months postoperatively compared to all other time points. There were no significant changes in gastrointestinal syndrome scores (Table 4).

**Table 3:** Serum nutrient concentrations preoperatively and at three, six, and 12-month follow-up In the "p-values" column, "Global" is the p-value for the overall association of the serum concentration with time. When the global p-value is <.05, p-values for all pairwise comparisons between time points with p<.05 are shown. SD=standard deviation, IQR=inter-quartile range, preop=preoperative, m=month

	Preop	3 months	6 months	12 months	p-values
Calcium (mg/dL)	n=92	n=48	n=48	n=44	Global: 0.0002
mean ± SD	9.4 ± 0.37	9.5 ± 0.38	9.4 ± 0.38	9.2 ± 0.39	Preop vs 12m: 0.048
range	8.5 - 10.5	8.9 - 10.8	8.2 - 10.3	8.5 - 10.2	3m vs 12m: <0.001
median (IQR)	9.3 (9.1 - 9.6)	9.5 (9.2 - 9.7)	9.4 (9.1 - 9.6)	9.2 (8.9 - 9.5)	6m vs 12m: 0.007
Vitamin B1 (nmol/L)	n=56	n=39	n=37	n=31	Global: 0.009
mean ± SD	113 ± 41.9	91.8 ± 36.6	102 ± 41.0	112 ± 32.6	Preop v 3m: 0.009
range	10 - 283	38 - 262	6 -243	55 - 191	3m vs 6m: 0.040
median (IQR)	110 (95 - 133)	86 (70 - 105)	99 (85 - 121)	102 (90 - 126)	3m vs 12m: 0.007
Vitamin B6 (nmol/L)	n=55	n=38	n=37	n=33	Global: 0.015
mean ± SD	24.3 ± 39.0	39.4 ± 87.7	22.7 ± 22.5	64.5 ± 117	Preop vs 12m: 0.002
range	2.2 - 221	1.2 - 387	2.8 - 124	2.5 - 499	3m vs 12m: 0.003
median (IQR)	10.5 (7 - 24)	11 (5 - 19)	21 (9 - 26)	28 (11 - 44)	6m v 12m: 0.009
Vitamin B12 (pg/mL)	n=90	n=41	n=43	n=35	0.064
mean ± SD	565 ± 349	565 ± 405	605 ± 479	778 ± 502	
range	121 - 1850	211 - 2000	185 - 2000	186 - 2000	
median (IQR)	444 (340 - 688)	446 (345 - 598)	388 (300 - 643)	659 (375 - 1040)	
Folate (ng/mL)	n=63	n=42	n=38	n=34	Global: 0.002
mean ± SD	13.4 ± 5.8	11.0 ± 6.2	10.6 ± 5.4	11.7 ± 6.0	Preop vs 3m: 0.007
range	3.3 - 24	2.2 - 24	2.7 - 20	3.8 - 22.7	Preop vs 6m: <0.001
median (IQR)	13 (9 - 19)	9 (7 - 16)	9 (7 - 15)	9 (7 - 18)	
Vitamin D total (ng/mL)	n=87	n=41	n=45	n=34	Global: <0.001
mean ± SD	24.6 ± 9.4	29.8 ± 11.6	33.1 ± 16.8	32.6 ± 17.6	Preop vs 3m: 0.012
range	6 - 58	4 - 67	12 - 103	10 - 95	Preop vs. 6m: <0.001
median (IQR)	25 (17 - 31)	30 (22 - 35)	30 (24 - 41)	29 (25 - 35)	Preop vs. 12m: <0.001
Zinc (ug/dL)	n=56	n=39	n=38	n=30	0.768
mean ± SD	77.2 ± 11.0	93.1 ± 93.3	77.1 ± 12.3	75.7 ± 16.1	
range	61 - 108	57 - 654	57 - 105	50 - 177	
median (IQR)	76 (69 - 84)	78 (68 - 86)	76 (65 - 85)	72 (68 - 83)	
Copper (ug/dL)	n=55	n=39	n=36	n=30	0.047
mean ± SD	137 ± 35.8	132 ± 33.9	130 ± 37.0	121 ± 26.4	Preop vs. 12m: 0.006
range	84 - 274	86 - 236	66 - 247	67 - 187	
median (IQR)	130 (113 - 152)	129 (109 - 147)	125 (108 - 142)	118 (102 - 130)	
Iron (ug/dL)	n=60	n=43	n=41	n=35	Global: 0.003
mean ± SD	64.8 ± 25.2	68.2 ± 22.1	65.3 ± 31.9	80.5 ± 31.1	Preop vs. 12m: <0.001
range	21 - 121	27 - 119	8 - 153	32 - 169	3m vs. 12m: 0.014
median (IQR)	66 (45 - 83)	66 (56 - 81)	65 (42 - 83)	81 (58 - 96)	6m vs. 12m: 0.002
Ferritin (ng/mL)	n=88	n=42	n=43	n=32	0.692
mean ± SD	77.8 ± 74.0	84.0 ± 105	79.3 ± 97.9	83.6 ± 83.0	
range	4 - 335	6 - 497	4 - 514	7 - 352	
median (IQR)	59 (24 - 107)	52 (21 - 99)	47 (22 - 105)	52 (23 - 120)	
TIBC (mcg/dL)	n=58	n=43	n=40	n=34	Global: <0.001
mean ± SD	362 ± 60.0	337 ± 54.7	350 ± 68.1	321 ± 71.5	Preop vs. 3m: <0.001
range	247 - 503	234 - 475	213 - 515	125 - 447	Preop vs. 6m: 0.044
median (IQR)	357 (321 - 391)	330 (298 - 375)	343 (313 - 394)	342 (285 - 371)	Preop vs 12m: <0.001

**Table 4:** Gastrointestinal syndrome response scores preoperatively and at three, six, and 12-month follow-up P-values represent the overall statistical test value. SD=standard deviation, IQR=inter-quartile range, preop=preoperative, m=month

	Preop	3 months	6 months	12 months	p values
Reflux syndrome	n=87	n=48	n=15	n=20	0.443
mean ± SD	0.36 ± 0.91	0.22 ± 0.54	0.42 ± 1.4	0.6 ± 1.4	
range	0 - 5	0 - 2	0 - 5.25	0 - 5	
median (IQR)	0 (0 - 0.38)	0 (0 - 0)	0 (0 - 0)	0 (0 - 0)	
Abdominal pain syndrome	n=87	n=48	n=15	n=20	0.658
mean ± SD	0.39 ± 0.56	0.32 ± 0.45	0.56 ± 0.64	0.50 ± 0.67	
range	0 - 3.7	0 - 1.7	0 - 2.3	0 - 2.3	
median (IQR)	0.33 (0 - 0.67)	0 (0 - 0.67)	0.33 (0 - 0.67)	0.17 (0 - 0.75)	
Indigestion syndrome	n=87	n=48	n=15	n=20	0.125
mean ± SD	0.43 ± 0.60	0.38 ± 0.49	0.82 ± 0.93	0.79 ± 0.88	
range	0 - 2.5	0 - 2.0	0 - 3.3	0 - 2.8	
median (IQR)	0.25 (0 - 0.5)	0.25 (0 - 0.5)	0.5 (0.13 - 1.1)	0.5 (0.19 - 1.0)	
Diarrhea syndrome	n=87	n=48	n=15	n=20	0.873
mean ± SD	0.35 ± 0.73	0.26 ± 0.87	0.42 ± 0.90	0.25 ± 0.72	
range	0 - 5.0	0 - 4.3	0 - 3.0	0 - 3.0	
median (IQR)	0 (0 - 0.5)	0 (0 - 0)	0 (0 - 0.33)	0 (0 - 0)	
Constipation syndrome	n=87	n=48	n=15	n=20	0.158
mean ± SD	0.43 ± 0.93	0.39 ± 0.68	0.49 ± 0.81	0.97 ± 1.4	
range	0 - 5.0	0 - 2.7	0 - 2.3	0 - 5.0	
median (IQR)	0 (0 - 0.33)	0 (0 - 0.67)	0 (0 - 0.5)	0.33 (0 - 1.8)	

## Discussion

Approximately one-third of participants who had data for all study labs one year after surgery displayed two or more nutritional deficiencies, the most common of which was vitamin D; however, serum vitamin D concentrations increased with the use of the patch. These results align with prior work as described above [7,14].

A more recent retrospective chart review by Saurabh et al. investigated the same multivitamin patch at the same endpoint as our study and included a comparator group (standard oral supplements) [17]. Their study population included 44 patients who underwent RYGB and had demographics (i.e., age, gender, and BMI) similar to participants in our study. In their study, 23.5% (n=17 in the patch group) of patients had two or more deficiencies one year after surgery. In our study, this was found to be 32%. However, our study examined additional nutrients (vitamin B6, copper, zinc, and iron), which may explain why we observed a greater number of deficiencies, particularly since vitamin B6 was the second most common deficiency. Both studies found vitamin D to be the most common deficiency; however, our study showed a significant increase in serum vitamin D concentrations (Table 2). Saurabh et al also reported a significant decrease in vitamin B1 serum concentrations after one year, but this was only observed at the three-month time point in our study. Of note, their study demonstrated a greater proportion of individuals presenting with nutritional deficiencies in the transdermal versus oral supplementation group. The addition of multiple time points in our study allowed for a demonstration of the abrupt effect on vitamin D deficiency versus the gradual effect on vitamin B6 deficiencies.

Given that approximately 40% of patients had vitamin D deficiency and 20% had vitamin B6 deficiency one year after surgery, these data suggest using only the patch for supplementation may not be adequate for these vitamins for some individuals but may suffice for the other nutrients. However, fewer vitamin D deficiencies and high serum concentrations were observed in patients after using the patch, which suggests partial or dose-dependent effectiveness. The absence of changes in GSRS syndrome scores suggests that patients do not develop side effects including constipation. However, it should be noted that the GSRS was validated in a population of patients with gastroesophageal reflux disease, not is a postoperative population [16]. Further advantages of the patch include increased oral intake due to decreased pill burden and others as outlined by Grammatikopoulou et al. [18].

The limitations of our study demand a cautious interpretation of the results. First, our study did not include a comparator group. Second, the absence of nutritional assessments (e.g., food frequency questionnaires and food diaries) leaves the possibility that our results were influenced by dietary variations. Furthermore, since this was a single-center study with primarily female participants, generalizability to other genders and geographical areas is limited. Lastly, there are many missing data, particularly at later time points, and the pattern of missing may not be random. Individuals may miss follow-up appointments more or less often than others based on the acuity of illness or socioeconomic factors not measured here. Those who are healthy may be more likely to forgo serial lab tests. Particularly during the last year of the study, patient contact with the healthcare system for routine follow-up was markedly decreased given the arrival of the coronavirus disease 2019 (COVID-19) pandemic.

## Conclusions

These data suggest that the PatchMD Multivitamin Plus patch may provide adequate supplementation of vitamins and minerals, with the exception of vitamins D and B6. Furthermore, the use of the patch does not appear to have associations with changes in gastrointestinal symptoms. However, randomized controlled trials are needed to verify these assumptions due to the various limitations discussed above. Current studies provide only observational data. Our data will be useful in designing appropriately powered studies to more formally assess these outcomes.
